# Crystal structure of a monoclinic polymorph of 5-amino-1,3,4-thia­diazol-2(3*H*)-one

**DOI:** 10.1107/S1600536814016055

**Published:** 2014-08-01

**Authors:** Namhun Kim, Sung Kwon Kang

**Affiliations:** a Department of Chemistry, Chungnam National University, Daejeon 305-764, Republic of Korea

**Keywords:** crystal structure, polymorph, thia­diazolone, hydrogen bonds

## Abstract

The title compound, C_2_H_3_N_3_OS, is a monoclinic (*P*2_1_/*c*) polymorph of the previously reported triclinic structure [Kang *et al.* (2012 ▶). *Acta Cryst.* E**68**, o1198]. The asymmetric unit contains two independent mol­ecules which are essentially planar, with r.m.s. deviations of 0.001 and 0.032 Å from the mean plane defined by the seven non-H atoms. In the crystal, N—H⋯N and N—H⋯O hydrogen bonds link the mol­ecules into a sheet parallel to (111).

## Related literature   

For structures and reactivity of thia­diazole derivatives, see: Parkanyi *et al.* (1989 ▶); Cho *et al.* (1996 ▶). For the triclinic polymorph, see; Kang *et al.* (2012 ▶).
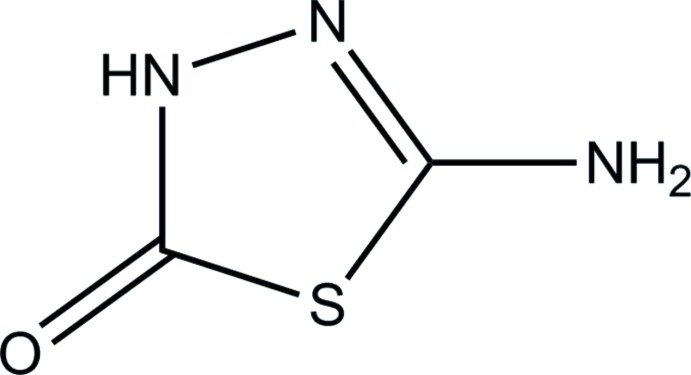


## Experimental   

### Crystal data  

C_2_H_3_N_3_OS
*M* *_r_* = 117.13
Monoclinic, 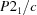

*a* = 3.8182 (3) Å
*b* = 10.8166 (7) Å
*c* = 21.8043 (15) Å
β = 91.015 (4)°
*V* = 900.37 (11) Å^3^
*Z* = 8
Mo *K*α radiation
μ = 0.58 mm^−1^
*T* = 296 K
0.21 × 0.1 × 0.09 mm

### Data collection  

Bruker SMART CCD area-detector diffractometer
Absorption correction: multi-scan (*SADABS*; Bruker, 2002 ▶) *T*_min_ = 0.911, *T*_max_ = 0.931
5812 measured reflections
1709 independent reflections
1376 reflections with *I* > 2σ(*I*)
*R*_int_ = 0.048

### Refinement  

*R*[*F* ^2^ > 2σ(*F* ^2^)] = 0.047
*wR*(*F* ^2^) = 0.099
*S* = 1.08
1709 reflections
151 parameters
All H-atom parameters refined
Δρ_max_ = 0.37 e Å^−3^
Δρ_min_ = −0.29 e Å^−3^

### 

Data collection: *SMART* (Bruker, 2002 ▶); cell refinement: *SAINT* (Bruker, 2002 ▶); data reduction: *SAINT*; program(s) used to solve structure: *SHELXS2013* (Sheldrick, 2008 ▶); program(s) used to refine structure: *SHELXL2013* (Sheldrick, 2008 ▶); molecular graphics: *ORTEP-3 for Windows* (Farrugia, 2012 ▶); software used to prepare material for publication: *WinGX* (Farrugia, 2012 ▶).

## Supplementary Material

Crystal structure: contains datablock(s) global, I. DOI: 10.1107/S1600536814016055/tk5326sup1.cif

Structure factors: contains datablock(s) I. DOI: 10.1107/S1600536814016055/tk5326Isup2.hkl

Supporting information file. DOI: 10.1107/S1600536814016055/tk5326Isup3.cml

**Fig. 1.**
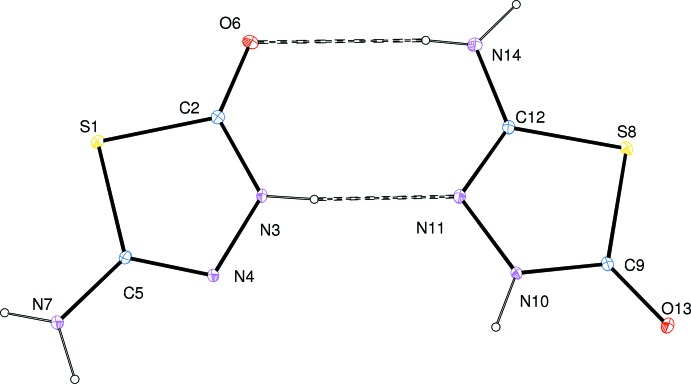
Molecular structure of the title compound, showing the atom-numbering scheme and 30% probability ellipsoids. Intermolecular N—H···N and N—H···O hydrogen bonds are indicated by dashed lines.

. DOI: 10.1107/S1600536814016055/tk5326fig1.tif
Mol­ecular structure of the title compound, showing the atom-numbering scheme and 30% probability ellipsoids. Inter­molecular N—H⋯N and N—H⋯O hydrogen bonds are indicated by dashed lines.

**Fig. 2.**
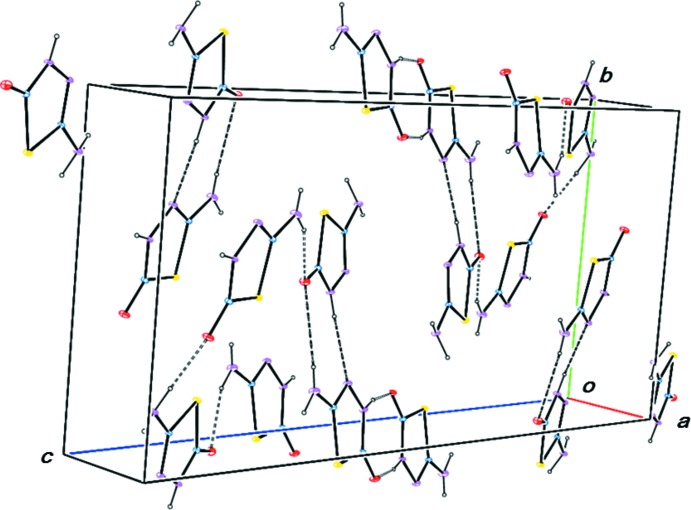
Part of the crystal structure of the title compound, showing molecules linked by intermolecular N—H···N and N—H···O hydrogen bonds (dashed lines).

. DOI: 10.1107/S1600536814016055/tk5326fig2.tif
Part of the crystal structure of the title compound, showing mol­ecules linked by inter­molecular N—H⋯N and N—H⋯O hydrogen bonds (dashed lines).

CCDC reference: 1013072

Additional supporting information:  crystallographic information; 3D view; checkCIF report

## Figures and Tables

**Table 1:** Hydrogen-bond geometry (Å, °)

*D*—H⋯*A*	*D*—H	H⋯*A*	*D*⋯*A*	*D*—H⋯*A*
N3—H3⋯N11	0.77 (3)	2.12 (3)	2.891 (4)	174 (3)
N7—H7*A*⋯O13^i^	0.86 (3)	2.07 (4)	2.913 (4)	167 (3)
N7—H7*B*⋯N4^ii^	0.85 (4)	2.21 (4)	3.048 (4)	171 (3)
N10—H10⋯O13^iii^	0.84 (3)	2.09 (3)	2.910 (3)	165 (3)
N14—H14*A*⋯O6^iv^	0.91 (4)	2.14 (4)	3.005 (4)	159 (4)
N14—H14*B*⋯O6	0.73 (4)	2.58 (4)	3.306 (5)	173 (4)

Symmetry codes: (i) 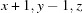; (ii) 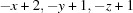; (iii) 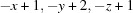; (iv) 
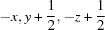
.

## supplementary crystallographic information

### S2. Structural commentary

5-Amino-2*H*-1,2,4-thia­diazo­lin-3-one heterocycle is an analog of cytosine (Parkanyi *et al.*, 1989). Derivatives of this heterocyclic compound are inter­esting in the anti­bacterial activity, potential carcinogenicity, and kinase inhibitor activity (Cho *et al.*, 1996). The title compound, 5-amino-1,3,4-thia­diazol-2(3*H*)-one (I) is an isomer of 5-amino-2*H*-1,2,4-thia­diazo­lin-3-one, which has become an attractive moiety due to potential biological activities. These heterocyclic compounds are potentially good ligands because of N, O, and S atoms which are good donor atoms to both transition metals (Cu, Zn, Cd) and lanthanide metals (Tb and Eu). In our inter­est to metal complexes with these heterocyclic compounds, the title compound was isolated accidently.

In (I), Fig. 1, two independent molecules comprise the asymmetric unit, which are linked by the inter­molecular N—H···N and N—H···O hydrogen bonds. The 1,3,4-thia­diazol-2-one units are almost planar, with r.m.s. deviations of 0.001 – 0.032 Å from the corresponding least-squares plane defined by the seven constituent atoms. The crystal structure is stabilized by the inter­molecular N—H···N and N—H···O hydrogen bonds, which link the molecules into a two-dimensional sheet parallel to *111* plane (Table 1 and Fig. 2).

### S5. Synthesis and crystallization

The title compound (I) was synthesized by the process of the previous report (Kang *et al.* 2012). Copper(II) chloride (1.36 g, 8 mmol) dissolved in ethanol, was added drop wise to a stirred ethano­lic solution containing 5-amino-1,3,4-thia­diazol-2(3*H*-one (1.87 g, 16 mmol). The mixture was stirred for 10 h at room temperature. The resulting solution was filtered and allowed to stand at room temperature. Colourless crystals of (I) were obtained at room temperature over a period of a few weeks.

### S6. Refinement

Crystal data, data collection and structure refinement details are summarized in Table 1. H atoms of the NH and NH_2_ groups were located in a difference Fourier map and refined freely [refined distances = 0.73 (4)–0.91 (4) Å].

### Crystal data

**Table:**

C_2_H_3_N_3_OS	*F*(000) = 480
*M**_r_* = 117.13	*D*_x_ = 1.728 Mg m^−^^3^
Monoclinic, *P*2_1_/*c*	Mo *K*α radiation, λ = 0.71073 Å
Hall symbol: -P 2ybc	Cell parameters from 1700 reflections
*a* = 3.8182 (3) Å	θ = 2.7–25.7°
*b* = 10.8166 (7) Å	µ = 0.58 mm^−^^1^
*c* = 21.8043 (15) Å	*T* = 296 K
β = 91.015 (4)°	Block, colourless
*V* = 900.37 (11) Å^3^	0.21 × 0.1 × 0.09 mm
*Z* = 8	

### Data collection

**Table:**

Bruker SMART CCD area-detector diffractometer	1376 reflections with *I* > 2σ(*I*)
Graphite monochromator	*R*_int_ = 0.048
φ and ω scans	θ_max_ = 25.8°, θ_min_ = 1.9°
Absorption correction: multi-scan (*SADABS*; Bruker, 2002)	*h* = −4→4
*T*_min_ = 0.911, *T*_max_ = 0.931	*k* = −12→13
5812 measured reflections	*l* = −26→26
1709 independent reflections	

### Refinement

**Table:**

Refinement on *F*^2^	0 restraints
Least-squares matrix: full	Hydrogen site location: difference Fourier map
*R*[*F*^2^ > 2σ(*F*^2^)] = 0.047	All H-atom parameters refined
*wR*(*F*^2^) = 0.099	*w* = 1/[σ^2^(*F*_o_^2^) + (0.0317*P*)^2^ + 0.833*P*] where *P* = (*F*_o_^2^ + 2*F*_c_^2^)/3
*S* = 1.08	(Δ/σ)_max_ < 0.001
1709 reflections	Δρ_max_ = 0.37 e Å^−^^3^
151 parameters	Δρ_min_ = −0.29 e Å^−^^3^

### Special details

**Table:**

Geometry. All e.s.d.'s (except the e.s.d. in the dihedral angle between two l.s. planes) are estimated using the full covariance matrix. The cell e.s.d.'s are taken into account individually in the estimation of e.s.d.'s in distances, angles and torsion angles; correlations between e.s.d.'s in cell parameters are only used when they are defined by crystal symmetry. An approximate (isotropic) treatment of cell e.s.d.'s is used for estimating e.s.d.'s involving l.s. planes.

### Fractional atomic coordinates and isotropic or equivalent isotropic displacement parameters (Å^2^)

**Table:**

	*x*	*y*	*z*	*U*_iso_*/*U*_eq_	
S1	0.5670 (2)	0.33458 (7)	0.36148 (3)	0.0316 (2)	
C2	0.4389 (8)	0.4860 (3)	0.33723 (13)	0.0297 (7)	
N3	0.5448 (8)	0.5631 (3)	0.38116 (12)	0.0327 (7)	
H3	0.487 (9)	0.631 (3)	0.3829 (14)	0.030 (10)*	
N4	0.7091 (7)	0.5177 (2)	0.43328 (11)	0.0301 (6)	
C5	0.7362 (8)	0.3992 (3)	0.42932 (13)	0.0259 (7)	
O6	0.2779 (7)	0.5107 (2)	0.28964 (10)	0.0454 (6)	
N7	0.8708 (8)	0.3270 (3)	0.47486 (13)	0.0349 (7)	
H7A	0.968 (9)	0.259 (3)	0.4641 (14)	0.035 (10)*	
H7B	0.983 (10)	0.364 (3)	0.5033 (17)	0.049 (11)*	
S8	0.0629 (2)	1.02405 (7)	0.34352 (4)	0.0334 (2)	
C9	0.2461 (8)	1.0171 (3)	0.41853 (13)	0.0294 (7)	
N10	0.3361 (7)	0.9003 (2)	0.42943 (12)	0.0315 (6)	
H10	0.438 (8)	0.884 (3)	0.4630 (14)	0.024 (8)*	
N11	0.2747 (8)	0.8126 (2)	0.38422 (11)	0.0359 (7)	
C12	0.1325 (8)	0.8645 (3)	0.33686 (13)	0.0296 (7)	
O13	0.2843 (7)	1.1052 (2)	0.45367 (10)	0.0458 (7)	
N14	0.0397 (10)	0.8045 (4)	0.28504 (14)	0.0502 (9)	
H14A	−0.052 (11)	0.853 (4)	0.2546 (19)	0.068 (13)*	
H14B	0.074 (10)	0.738 (4)	0.2852 (17)	0.043 (12)*	

### Atomic displacement parameters (Å^2^)

**Table:**

	*U*^11^	*U*^22^	*U*^33^	*U*^12^	*U*^13^	*U*^23^
S1	0.0410 (5)	0.0227 (4)	0.0307 (4)	0.0018 (4)	−0.0078 (3)	−0.0059 (3)
C2	0.0324 (18)	0.0272 (17)	0.0295 (16)	0.0007 (14)	−0.0021 (13)	−0.0011 (13)
N3	0.0497 (19)	0.0174 (14)	0.0307 (15)	0.0063 (13)	−0.0095 (12)	−0.0019 (11)
N4	0.0401 (16)	0.0220 (14)	0.0278 (13)	0.0087 (12)	−0.0088 (11)	−0.0033 (11)
C5	0.0287 (17)	0.0221 (16)	0.0269 (15)	0.0020 (13)	0.0001 (12)	−0.0042 (12)
O6	0.0609 (17)	0.0390 (14)	0.0355 (13)	0.0025 (12)	−0.0201 (12)	0.0028 (11)
N7	0.0482 (19)	0.0240 (16)	0.0319 (15)	0.0073 (14)	−0.0118 (13)	−0.0026 (13)
S8	0.0422 (5)	0.0250 (4)	0.0326 (4)	0.0077 (4)	−0.0091 (3)	0.0031 (3)
C9	0.0343 (18)	0.0242 (16)	0.0296 (16)	0.0068 (14)	−0.0037 (13)	0.0004 (13)
N10	0.0479 (18)	0.0233 (14)	0.0229 (13)	0.0116 (12)	−0.0093 (12)	−0.0023 (11)
N11	0.0550 (19)	0.0239 (14)	0.0284 (14)	0.0093 (13)	−0.0091 (12)	−0.0033 (11)
C12	0.0358 (19)	0.0242 (16)	0.0288 (16)	0.0051 (14)	−0.0027 (13)	−0.0019 (13)
O13	0.0714 (18)	0.0255 (13)	0.0400 (13)	0.0124 (12)	−0.0154 (12)	−0.0079 (11)
N14	0.079 (3)	0.036 (2)	0.0346 (18)	0.0114 (18)	−0.0230 (16)	−0.0056 (15)

### Geometric parameters (Å, º)

**Table:**

S1—C5	1.749 (3)	S8—C12	1.753 (3)
S1—C2	1.786 (3)	S8—C9	1.769 (3)
C2—O6	1.226 (4)	C9—O13	1.230 (3)
C2—N3	1.328 (4)	C9—N10	1.329 (4)
N3—N4	1.379 (3)	N10—N11	1.385 (3)
N3—H3	0.77 (3)	N10—H10	0.84 (3)
N4—C5	1.289 (4)	N11—C12	1.287 (4)
C5—N7	1.357 (4)	C12—N14	1.345 (4)
N7—H7A	0.86 (3)	N14—H14A	0.91 (4)
N7—H7B	0.85 (4)	N14—H14B	0.73 (4)
			
C5—S1—C2	88.81 (14)	C12—S8—C9	88.66 (14)
O6—C2—N3	127.9 (3)	O13—C9—N10	126.7 (3)
O6—C2—S1	125.5 (2)	O13—C9—S8	125.7 (2)
N3—C2—S1	106.5 (2)	N10—C9—S8	107.6 (2)
C2—N3—N4	120.0 (3)	C9—N10—N11	118.9 (3)
C2—N3—H3	123 (2)	C9—N10—H10	118 (2)
N4—N3—H3	115 (2)	N11—N10—H10	123 (2)
C5—N4—N3	109.5 (2)	C12—N11—N10	109.6 (2)
N4—C5—N7	123.6 (3)	N11—C12—N14	124.4 (3)
N4—C5—S1	115.1 (2)	N11—C12—S8	115.2 (2)
N7—C5—S1	121.2 (2)	N14—C12—S8	120.4 (3)
C5—N7—H7A	117 (2)	C12—N14—H14A	115 (3)
C5—N7—H7B	116 (2)	C12—N14—H14B	115 (3)
H7A—N7—H7B	113 (3)	H14A—N14—H14B	130 (4)

### Hydrogen-bond geometry (Å, º)

**Table:**

*D*—H···*A*	*D*—H	H···*A*	*D*···*A*	*D*—H···*A*
N3—H3···N11	0.77 (3)	2.12 (3)	2.891 (4)	174 (3)
N7—H7*A*···O13^i^	0.86 (3)	2.07 (4)	2.913 (4)	167 (3)
N7—H7*B*···N4^ii^	0.85 (4)	2.21 (4)	3.048 (4)	171 (3)
N10—H10···O13^iii^	0.84 (3)	2.09 (3)	2.910 (3)	165 (3)
N14—H14*A*···O6^iv^	0.91 (4)	2.14 (4)	3.005 (4)	159 (4)
N14—H14*B*···O6	0.73 (4)	2.58 (4)	3.306 (5)	173 (4)

Symmetry codes: (i) *x*+1, *y*−1, *z*; (ii) −*x*+2, −*y*+1, −*z*+1; (iii) −*x*+1, −*y*+2, −*z*+1; (iv) −*x*, *y*+1/2, −*z*+1/2.
