# Supplementary figures and images for: Crystal structure of a monoclinic polymorph of 5-amino-1,3,4-thia­diazol-2(3H)-one
Source: Acta Crystallogr Sect E Struct Rep Online. 2014 Aug 1;70(Pt 9):o922. doi: 10.1107/S1600536814016055 (PMC4186073; doi:10.1107/S1600536814016055)

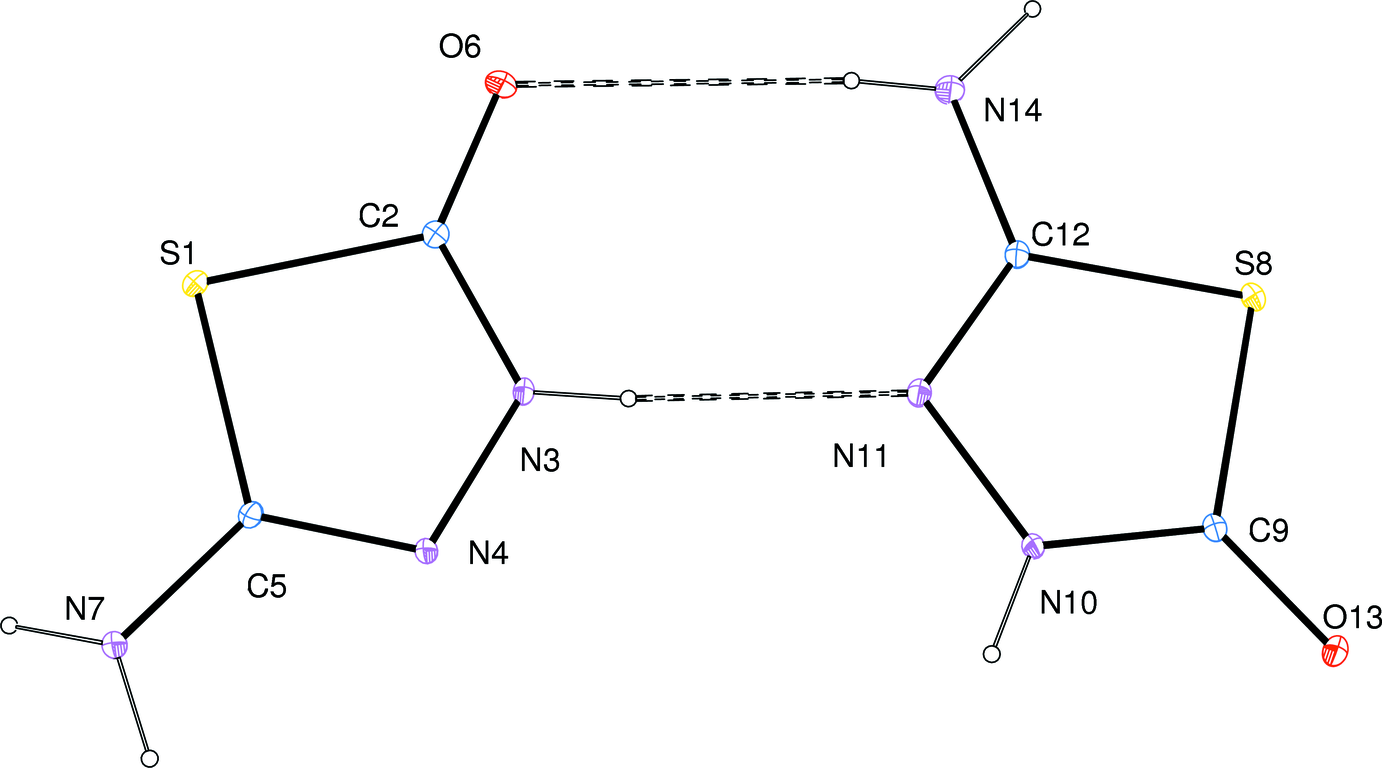

Supplement: Supplementary file 4 [file e-70-0o922-fig1.tif]

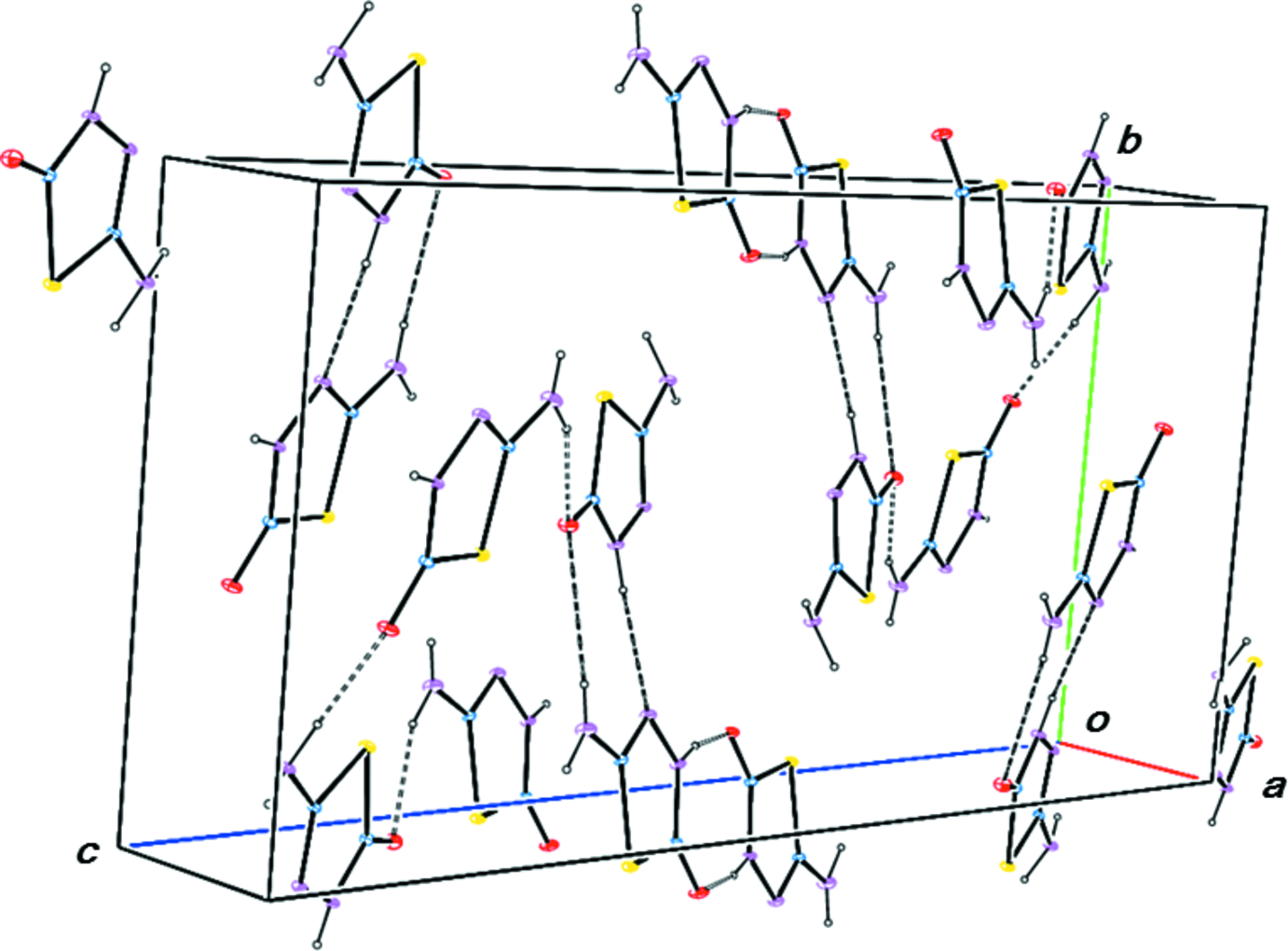

Supplement: Supplementary file 5 [file e-70-0o922-fig2.tif]
